# Variability in the Response of Bacterial Community Assembly to Environmental Selection and Biotic Factors Depends on the Immigrated Bacteria, as Revealed by a Soil Microcosm Experiment

**DOI:** 10.1128/mSystems.00496-19

**Published:** 2019-12-03

**Authors:** Xiaogang Wu, Yun Wang, Ying Zhu, Hao Tian, Xianchao Qin, Changzheng Cui, Liping Zhao, Pascal Simonet, Xiaojun Zhang

**Affiliations:** a State Key Laboratory of Microbial Metabolism, School of Life Sciences & Biotechnology, Shanghai Jiao Tong University, Shanghai, China; b Joint International Research Laboratory of Metabolic & Developmental Sciences, School of Life Sciences & Biotechnology, Shanghai Jiao Tong University, Shanghai, China; c Environmental Microbial Genomics Group, Laboratoire AMPERE, CNRS, Ecole Centrale de Lyon, Université de Lyon, Ecully, France; d School of Resource and Environmental Engineering, East China University of Science and Technology, Shanghai, China; Lawrence Berkeley National Laboratory

**Keywords:** bacterial community, community assembly, microcosms, selection, sterile soil

## Abstract

Exploring the assembly mechanism of microbiota is critical for understanding soil ecosystem functions. However, the relative importance of different biotic and abiotic factors in determining the bacterial community has not been properly clarified. In this study, the effects of inocula and recipients on the assembly of the soil community were investigated to evaluate their importance by inoculation experiments with sterile soil. Two distinct soils, conventional nitrogen-fertilized soil and aromatic-compound-contaminated soil, were sterilized, cross inoculated, and incubated for 2 months under different inoculation doses and oxygen conditions. The results showed that the greatest variation in community structure emerged in the samples inoculated with distinct inocula rather than in the samples of different soil recipients. The phylogenies in the two inocula were diverse and dissimilar, although there were many ecologically equivalent bacteria. When the inocula with dissimilar ecologically equivalent species were used, the assembled communities were primarily determined by the inocula as indicated by the beta diversity and variation partitioning analyses. In contrast, environmental selection dominated the process when ecologically equivalent species in the inocula were similar, as when only one type of inoculum was used, where the soil habitat selected the most adaptive bacteria from the defined inoculum pool. These results indicate that inoculated bacteria are dominant over environmental selection if they are sufficiently dissimilar, although the effect of environmental selection is more obvious when similar bacteria are inoculated in the soil for community assembly. Our findings suggest that the immigration of exotic bacteria could be a primary factor impacting community assembly.

**IMPORTANCE** The soil microbiota conducts important biological ecosystem functions, but the mechanism underlying community-environment interactions for soil microbiota remains unclear. By using two distinct soils for cross inoculation, we successfully simulated the assembly of the bacterial community in sterile soil. Thus, the reasons why inoculum and recipient have dominated community assembly in previous investigations are investigated in this study. We found that inoculated bacteria presided over environmental selection for community assembly due to the varied difference of ecological equivalent bacteria, either divergent or convergent. The significance of neutrality for the ecologically equivalent bacterial species that immigrated into the recipients should be emphasized in exploring the mechanisms of community assembly. Our finding is helpful for understanding the community-environment interaction, a basic question in ecology, and it would shed light on this issue that has perplexed scientists for many years.

## Abstract (precis)

The soil microbiota conducts important biological ecosystem functions, but the mechanism underlying community-environment interactions for soil microbiota remains unclear. By using two distinct soils for cross inoculation, we successfully simulated the assembly of the bacterial community in sterile soil. Thus, the reasons why inoculum and recipient have dominated community assembly in previous investigations are investigated in this study. We found that inoculated bacteria presided over environmental selection for community assembly due to the varied difference of ecological equivalent bacteria, either divergent or convergent. The significance of neutrality for the ecologically equivalent bacterial species that immigrated into the recipients should be emphasized in exploring the mechanisms of community assembly. Our finding is helpful for understanding the community-environment interaction, a basic question in ecology, and it would shed light on this issue that has perplexed scientists for many years.

## INTRODUCTION

The soil environment harbors the greatest bacterial diversity (1). These bacteria play critical roles in numerous biological functions, including soil fertility regulation, pollutant degradation, plant health, and global cycling of carbon, nitrogen, and other nutrients (2). Understanding soil biodiversity and ecosystem function is critical for deciphering the mechanisms that sustain the assembly of different soil bacterial populations into functional communities and planning management strategies for their activities further to improve ecosystem functions. Thus far, the mechanisms underlying the generation of bacterial communities are not clear and require further study. For example, immigration of bacteria and environmental selection are both reported as important ecological factors in determining community assembly (3–5), although their relative importance is not consistent among the published references (4–7).

The community-environment interaction has been extensively studied in community ecology (8). Some authors claim that environmental selection is extremely important for structuring microbiota and that the community structures in each niche are primarily determined through deterministic partitioning of resources among organisms (9–11). Habitat and environmental filtering perspectives suggest that community assembly is a specific and predicable process (12–14). For example, the famous statement proposed by Baas Becking is that “everything is everywhere, the environment selects.” Thus, there might be a one-to-one match between niche availability (or environmental condition) and community structure. In soil environments, the soil type in particular is critical for determining the structures of bacterial communities (15). This conclusion was supported by Griffiths et al. (16), who seeded sterilized sandy and clay soil samples with soil bacterial communities and found that the structures of the newly developed microbiota were largely influenced by the soil type, rather than the initial inoculum. Similar conclusions were drawn by another group, who used two soils sampled from England and Italy that were sterilized, reinoculated, and incubated prior to DNA extraction and sequencing (17), and by Xun et al. (5), who developed the same experiment with two soil samples from the same origin but differing fertilization treatments.

However, other studies have reported that the role of biotic factors is more important for structuring the microbiota. According to the neutral theory, the influence of environmental factors on each species in the habitat is neutral and neither promotes nor inhibits change (18–20). Stochastic factors, such as immigration, dispersal birth/death processes, drift, and speciation, are the primary drivers of ecological diversity and community structure (20). This theory has been reported to accurately predict the future structures of bacterial communities (21, 22). Empirical evidence has demonstrated that the local species pool controls community composition due to dispersal limitations for bacteria, which is consistent with the neutral process (19, 23). Thus, microcosm experiments using sterilized media recolonized by microbes provide a suitable system for examining the importance of inocula versus environmental factors for the initial assembly of bacterial communities (24). In these experiments, several natural microbe sources are seeded as inocula into sterilized environmental samples and incubated under laboratory conditions to elucidate the resulting community assembly and the factors that affect it (4, 6, 7). Langenheder et al. (6) performed batch culture experiments in which bacteria from eight distinct aquatic habitats were regrown under identical conditions to determine the extent to which similar communities would develop under the same selective pressures. These results demonstrated that the inocula had a greater impact than the environmental conditions (6). Pagaling et al. (7) drew the same conclusions from their experiments with sediment and water samples collected from seven sources colonized by significantly different bacterial communities. The taxonomic structure of the developed bacterial communities in the incubated microcosms clearly demonstrated source dependence (7). The results were not due to the laboratory effect because the same assembly theory was applied under field conditions. Different sterilized liquid growth media processed into microcosms were spread over nine field locations across three spatial scales in the Inner Mongolian grassland to be naturally reinoculated by indigenous bacteria. The structures of the bacterial communities that assembled in the microcosms were dictated by the different inocula instead of by the growth media due to their separate field locations and dissimilar immigration of species (4).

In general, inoculum and recipient dominated community assembly in previous investigations. However, the variability in all investigations has perplexed scientists and obscured community assembly mechanisms. From these varied conclusions arose the question of what is the relative importance of inoculum and recipient in the determination of community structure? To answer this question, in this study, we simulated the bacterial community assembly in soil by incubating microcosms of sterilized soil reinoculated with two distinct soil samples. The impacts of the inoculation dosage and aeration condition were also checked. After incubation, microbiota analyses were performed to determine the relative importance of inocula and recipient conditions when comparing all developed microbiota derived from the different soil inocula versus separately comparing the microbiota developed from each inoculum.

## RESULTS

### Changes in soil biomass after incubation.

**FIG 1:**
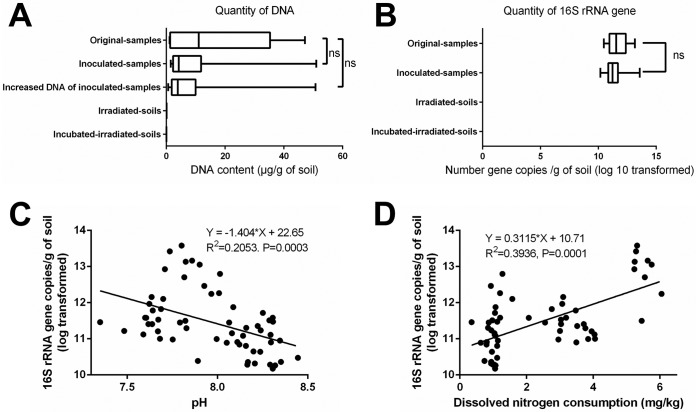
Recovery of bacterial biomass after incubation. (A) Box plot reflecting the quantity of extracted DNA from soil samples. Increased DNA of inoculated samples is the weight of recovered DNA from the sample after incubation minus the weight of DNA introduced by the inoculum. (B) Box plot reflecting the quantity of 16S RNA gene copies within soil. The quantity in the irradiated soil was under the detection limit (negative). (C) Relationship between pH and 16S rRNA gene copies (log transformed). (D) Relationship between dissolved nitrogen (DN) consumption and 16S rRNA gene copies (log transformed). The box plots represent the smallest observation, lower quartile, median, upper quartile, and largest observation. Values are not significantly different (ns) according to the Mann-Whitney U test (nonparametric test). Linear regression was used to describe the relationships among pH, DN consumption, and bacterial abundance.

Regular plating of the uninoculated sterilized soil samples on growth media did not yield bacterial or fungal colonies, which confirmed the effectiveness of the irradiation treatment used to kill the indigenous microorganisms. Additionally, the effectiveness of irradiation was monitored by measuring the amount of DNA extracted from the soil samples. DNA quantities of 1.45 and 32.90 μg · g^−1^ soil were obtained from the aromatic-contaminated soil (ACS) and optimized nitrogen-fertilized soil (ONS), respectively, before irradiation and decreased to 0.084 and 0.098 μg · g^−1^ after irradiation, thus confirming the strong lethal effect and denaturation of most of the DNA (Fig. 1A). Persisting extracellular DNA was gradually degraded until completely eliminated by DNase enzymes during the incubation. The decrease in DNA and the lack of microorganisms growing from the microcosms excluded the possibility that any indigenous microorganisms survived the sterilization treatment (Fig. 1A).

Bacterial growth in the inoculated samples was assessed by quantifying the total amount of DNA and the 16S rRNA gene copy numbers (see Table S1 in the supplemental material). These two parameters indicated the recovery of the microbiota after 2 months of incubation at a quantitative level similar to the level detected in the samples prior to sterilization (*P* = 0.1707 for DNA content, *P* = 0.8404 for increased DNA, and *P* = 0.0694 for 16S rRNA gene copies by the Mann-Whitney U test) (Fig. 1A and B). In addition, the growth for the total extracted DNA and the 16S rRNA gene copy number reached a plateau, confirming that the microbiota reached its biotic capacity in both soils within 2 months (Fig. 1A and B). The numbers of bacterial cells (abundance) that developed from a subsample inoculated into previously sterilized soil samples were higher under low pH than under high pH conditions (Fig. 1C, *R*^2^ = 0.2053, *P* = 0.0003) and under high dissolved nitrogen (DN) consumption than under low DN consumption conditions (Fig. 1D, *R*^2^ = 0.3936, *P* = 0.0001).

10.1128/mSystems.00496-19.2 TABLE S1 Quantity of DNA and number of 16S rRNA gene copies in different treatments. Download Table S1, PDF file, 0.1 MB. Copyright © 2019 Wu et al. 2019 Wu et al. This content is distributed under the terms of the Creative Commons Attribution 4.0 International license.

### Overall structural shift of bacterial communities after incubation.

**FIG 2:**
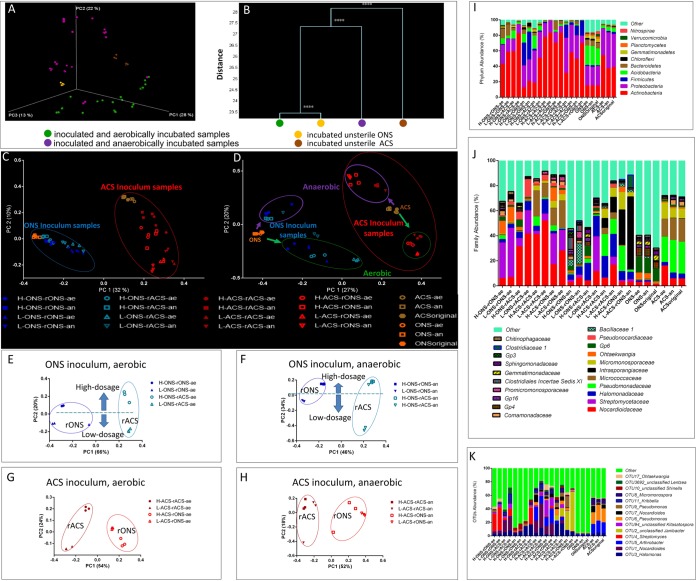
Long-term soil incubation shows significant inoculum-associated and environment-associated structural segregation of the bacterial community. (A) Bray-Curtis principal-coordinate analysis (PCoA) of the soil bacterial community structure based on the family data. (B) Clustering of the soil microbiota based on Bray-Curtis distances calculated with multivariate analysis of variance (MANOVA). Values that are significantly different (*P* < 0.0001) are indicated by four asterisks. (C) Unweighted UniFrac PCoA of the soil bacterial community structure based on operational taxonomic unit (OTU) data. (D) Bray-Curtis PCoA of the soil bacterial community structure based on OTU data. (E) Bray-Curtis PCoA of the ONS inoculum bacterial community structure under aerobic conditions based on OTU data. (F) Bray-Curtis PCoA of the ONS inoculum sample bacterial community structure under anaerobic conditions based on OTU data. (G) Bray-Curtis PCoA of the ACS inoculum sample bacterial community structure under aerobic conditions based on OTU data. (H) Bray-Curtis PCoA of the ACS inoculum sample bacterial community structure under anaerobic conditions based on OTU data. MANOVA analysis showed significant differences between the circled clusters (*P* < 0.0001). (I) Phylum-level community structures after the different treatments. The distributions presented at different levels are based on the 80% similarity clusters of the OTUs. (J) Family-level community structures of the different treatments. The distributions presented at different levels are based on the 80% similarity clusters of the OTUs. (K) OTU-level community structures of the different treatments recovered from sterile soil. Two sterilized soils (rONS and rACS) were inoculated with a subsample of their initial inoculum and a subsample from the other soil (ONS and ACS) with different inoculation doses (heavy [H] and light [L]) and oxygen conditions (aerobic [ae] and anaerobic [an]). In all PCoA plots, the percentage of the variation explained by the plotted principal coordinates (PCs) is shown in parentheses.

A total of 4,214,140 quality-controlled sequences for the amplicons of the amplified V3-V4 region of the 16S rRNA gene were obtained (median, 47,058 sequences; range, 20,379 to 88,416 sequences). These sequences were clustered into 5,338 OTUs (operational taxonomy units) (97% similarity). Rarefaction and Shannon diversity curves revealed that the ONS-inoculated samples had higher diversity (alpha diversity index) than the ACS-inoculated samples (see Fig. S1 and Table S2 in the supplemental material). The aeration conditions significantly influenced the bacterial communities that developed from the inocula in the samples of sterilized soil. The MANOVA (multivariate analysis of variance) test separated the resulting bacterial communities into two groups according to aerobic or anaerobic incubation status (Fig. 2A and B). This result was confirmed by the Bray-Curtis principal-coordinate analysis (PCoA) based on the OTU data, which showed divergent development of the bacterial communities driven by aeration during incubation (Fig. 2D).

10.1128/mSystems.00496-19.1 FIG S1 Bacterial alpha diversity of the soil samples. (A) Rarefaction analysis. Repeated sampling of OTU subsets was used to evaluate where further sampling would likely yield additional taxa, as indicated by whether the curve has reached a plateau value. (B) Shannon diversity index curves to estimate the diversity of taxa present in individual soil samples. (C) Comparison of observed OTUs between ONS-inoculated samples and ACS-inoculated samples. (D) Comparison of the Shannon diversity index between ONS-inoculated samples and ACS-inoculated samples. The Mann-Whitney U test (nonparametric test) was used to analyze the variation between two groups. ****, *P* < 0.0001. (E) Venn diagram of the OTU distribution between two groups of differently inoculated samples. Download FIG S1, TIF file, 3.0 MB. Copyright © 2019 Wu et al. 2019 Wu et al. This content is distributed under the terms of the Creative Commons Attribution 4.0 International license.

10.1128/mSystems.00496-19.3 TABLE S2 Alpha diversity indices of different treatments after incubation for 2 months. Download Table S2, PDF file, 0.1 MB. Copyright © 2019 Wu et al. 2019 Wu et al. This content is distributed under the terms of the Creative Commons Attribution 4.0 International license.

However, the inoculum was another factor that contributed to the taxonomic structuration of the soil microbiota that developed in the soil microcosms, as evidenced by the significant differences in the MANOVA between the ONS- and ACS-inoculated samples detected by the unweighted UniFrac PCoA plots for the OTUs (Fig. 2C). The results also showed that the samples separated according to the recipient type along the principal component 1 (PC1) axis and according to the inoculum dosage along the PC2 axis when the microcosms were seeded with different amounts of the same inoculum (Fig. 2E to H). MANOVA showed significant differences between the recipients, which indicated a recipient effect on the community profile when the same inoculum was inoculated with both high and low inoculum dosage and incubated under aerobic and anaerobic conditions.

### Bacterial community structuration in soil microcosms.

Compared with the unsterilized soil microcosms, the inoculated sterile soil developed significantly different bacterial communities depending on the inoculum, recipient, and incubation conditions. Aerobic incubation of the microcosms led to systematic development of *Actinobacteria*, particularly representatives of *Nocardioidaceae* and *Streptomycetaceae*, which were found at higher quantitative levels than those observed in the controls, regardless of the inoculum used. When the microcosms were incubated under anaerobic conditions, the proportions of *Firmicutes* and *Proteobacteria* increased significantly, with a particularly significant increase in representatives of *Halomonadaceae* (Fig. 2I and J).

However, the taxonomic analysis also indicated that the two inocula differentially impacted the bacterial community structure of the developed microbiota. At the phylum level, the microbiota was dominated by *Acidobacteria* when the microcosms were inoculated with the ONS inoculum (Fig. 2I). An impact of the inoculum was also noted at the family level when the microcosms were incubated anaerobically, with a prevalence of Bacillaceae 1 and Clostridiales_Incertae Sedis XI (both *Firmicutes*) in the samples with the ONS samples being used as inoculum and a prevalence of *Micromonosporaceae* (*Actinobacteria*) for these samples in which the ACS was used as inoculum (Fig. 2J).

The development of bacterial communities in previously sterilized soils was impacted less by the physical and chemical characteristics of the recipient soils than by the inoculum or the incubation conditions, including aeration (Fig. 2K). The resulting community structure suggested that the species influenced by the recipient type also depended on the inoculum, with several species specifically enriched in samples according to the inoculum used. For instance, after inoculation with the ONS inoculum, we detected 2,390 OTUs in the ONS-inoculated samples, whereas 801 OTUs were detected in the ACS-inoculated samples (Fig. S1). Several OTUs were positively influenced by the recipient soils when the ONS inoculum was used, including OTU4 (*Streptomyces*) and OTU17 (*Ohtaekwangia*), which were specifically enriched by the combination of ONS as the donor and recipient (H-ONS-rONS-ae and L-ONS-rONS-ae) (treatments explained in “Soil microcosms” in Materials and Methods), and OTU11 (*Kribbella*), which was enriched by the combination of ONS as the donor and ACS as the recipient (H-ONS-rACS-ae and L-ONS-rACS-ae). The relative abundance of OTU_3 (*Halomonas*) depended on a recipient soil with a higher proportion of ACS (H-ONS-rACS-an and L-ONS-rACS-an) than ONS (H-ONS-rONS-an and L-ONS-rONS-an). Several OTUs were positively quantitatively influenced by the recipient soil when the ACS inoculum was used, including OTU1 (*Nocardioides*) and OTU11 (*Kribbella*), which were enriched in H-ACS-rACS-ae and L-ACS-rACS-ae. OTU94 (unclassified *Kitasatospora*), OTU4 (*Streptomyces*), and OTU17 (*Ohtaekwangia*) were enriched in H-ACS-rONS-ae and L-ACS-rONS-ae. OTU3 (*Halomonas*) was more abundant in H-ACS-rACS-an and L-ACS-rACS-an. OTU2 (unclassified *Janibacter*) was enriched in H-ACS-rONS-an and L-ACS-rONS-an (Fig. 2K).

### Impact of biotic and abiotic factors on shaping of soil bacterial communities.

**FIG 3:**
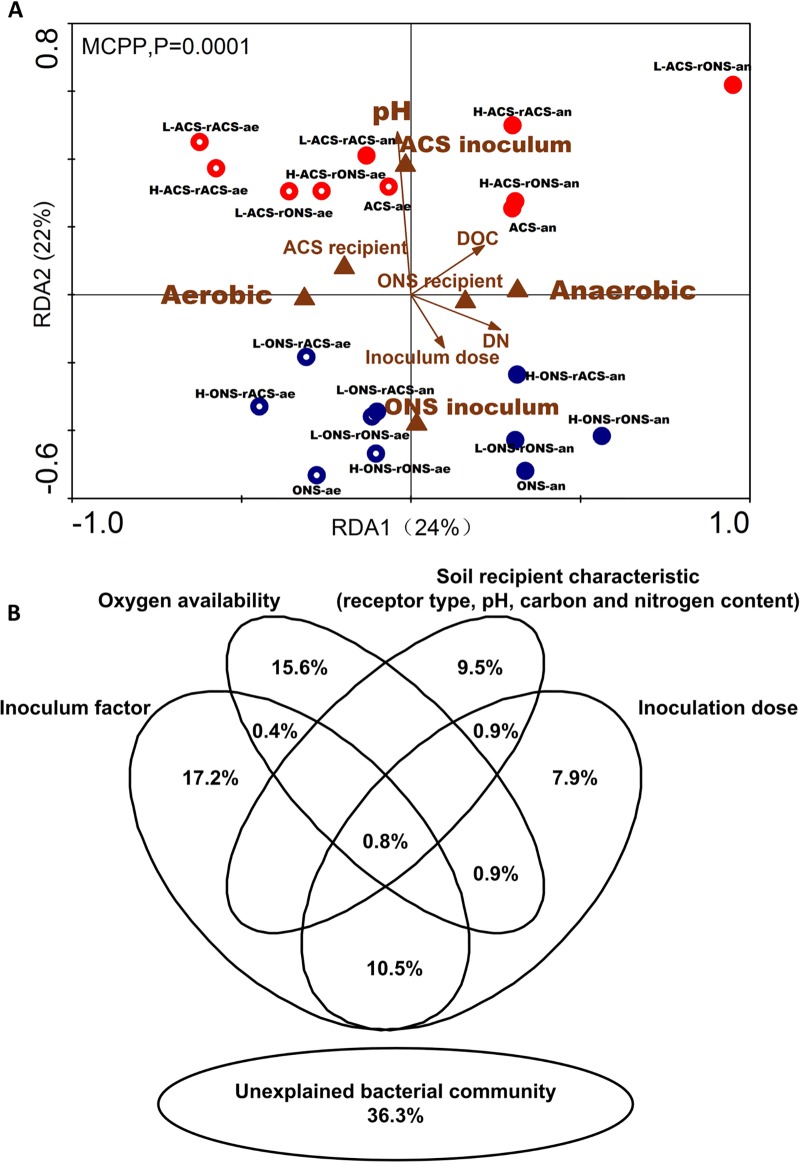
Ecological effects of biotic and abiotic factors on bacterial communities. (A) Redundancy analysis (RDA) plot of the microbiota composition resulting from different treatments based on the sequencing data. Nonnominal environmental variables are indicated by brown arrows, and nominal environmental variables are represented by brown triangles. Different management strategies are represented by circles. Blue circles represent ONS inoculum samples, and red circles represent ACS inoculum samples. Circles containing small white circles represent aerobic samples, and solid circles represent anaerobic samples. (B) Variation partitioning analysis (VPA) was used to determine the effects of the inoculum factor, oxygen availability, soil basic characteristics, receptor and dosage, and interactions between these parameters on the bacterial community structure. The Venn diagram shows the percentage of variation explained by the factors alone and in combination. The unexplained variation is depicted as an ellipse at the bottom of panel B.

Redundancy analysis (RDA) (Fig. 3A) assessed three pairs of nominal variables (oxygen condition, inoculum, and recipient soil) and four nonnominal variables (inoculation dose, pH, dissolved organic carbon [DOC], and DN) demonstrated that the bacterial community composition was significantly (*r* = 0.887, *P* = 0.0001) shaped by several key soil environmental variables. Bacterial communities were well separated by RDA1 (24%) according to the major variable oxygen condition and by RDA2 (22%) according to the major variable inoculum. However, RDA did not show clear clustering of the soil samples based on the recipient soils.

A variation partitioning analysis (VPA) was performed to calculate the relative contributions of the inoculum (type and dosage), oxygen, and recipient basic soil characteristics on the taxonomic composition of the bacterial community after 2 months of incubation (Fig. 3B). These variables explained 63.7% of the observed variation, with inoculum type accounting for the largest proportion (17.2%; *P* < 0.001), followed by oxygen availability (15.6%; *P* < 0.001). The characteristics of the recipient soil, including the type of recipient soil, explained 9.5% of the observed variation (*P* < 0.001), followed by the inoculum density (dosage) (7.9%; *P* < 0.001).

### Correlation of OTUs with the inoculum.

**FIG 4:**
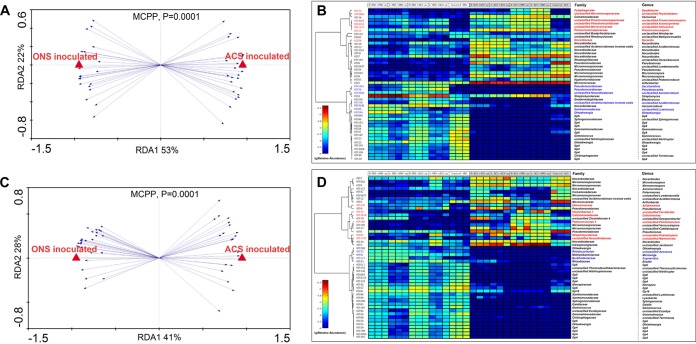
Redundancy analysis (RDA) plot of the bacterial community composition determined by the inoculum factor and heat map of the RDA-identified key OTUs responding to the inoculum factor. The inoculum sources were used as environmental variables. OTUs are indicated by blue arrows. *P* values were obtained by the Monte Carlo permutation procedure (MCPP) (*P* = 0.0001). (A) Aerobic conditions. (C) Anaerobic conditions. (B and D) Key OTUs responding to the inoculum factor under aerobic (B) and anaerobic (D) incubation. The color of the spot corresponds to the log-transformed relative abundance of the OTU. The OTUs are organized according to Spearman’s correlation of the OTU abundance. The family and genus names of the OTUs are shown to the right. OTUs whose names are in red in panels B and D are rare species in the control ACS sample but enriched in the ACS inoculum sample. OTUs whose names are in blue in panels B and D are rare species in the control ONS sample but enriched in the ONS inoculum sample.

The RDA approach was used to identify the OTUs driven by the inoculum and to determine the main contributor to the establishment of the community structure in the incubated soil samples (Fig. 4A and C). Under aerobic incubation conditions, 46 significantly different OTUs were found in the developed bacterial communities inoculated with either the ONS or ACS subsamples (Fig. 4A and B). Twenty-three OTUs were enriched following inoculation with ONS and were affiliated with 15 different genera, and 23 OTUs were enriched after the microcosms were inoculated with ACS and affiliated with 17 different genera. Under anaerobic incubation conditions, the number of different OTUs in the developed bacterial communities according to the inoculum type was 49 (RDA selection, Fig. 4C and D), with 28 OTUs enriched in the ONS-inoculated samples affiliated with 19 different genera, including 5 OTUs in *Gp4* and *Gp6*. The 21 OTUs enriched in the ACS-inoculated samples were affiliated with 18 different genera, with the 3 OTUs with the highest abundance belonging to *Pseudomonas*.

Interestingly, all of these bacteria, which could be distinguished by the inoculum and aeration conditions, behaved (with regard to growth and survival) similarly regardless of the recipient soil. Furthermore, among the OTUs correlated with the inoculum factor, most were abundant in the initial inoculum (with a relative abundance of >0.1% in the original soil), whereas few of them were rare (with a relative abundance of <0.1% in the original soil). All of these OTUs were enriched after incubation, and some became predominant after incubation, including OTU74 (*Dyadobacter*), OTU5094 (unclassified *Phytohabitans*), OTU4024 (unclassified *Promicromonospora*), OTU4151 (unclassified *Actinosynnema*), OTU1177 (unclassified *Citricoccus*), OTU13 (*Promicromonospora*), OTU59 (*Nocardia*), OTU1651 (*Saccharothrix*), OTU70 (*Pseudonocardia*), OTU3050 (unclassified *Aeromicrobium*), OTU156 (unclassified *Aciditerrimonas*), OTU86 (unclassified *Luteimonas*), OTU395 (*Ohtaekwangia*), OTU125 (*Aliidiomarina*), OTU15 (unclassified *Fervidicella*), OTU1610 (*Cellulomonas*), OTU103 (unclassified *Pelotomaculum*), OTU94 (unclassified *Kitasatospora*), OTU1372 (unclassified *Marmoricola*), OTU72 (unclassified *Azonexus*), and OTU117 (*Cupriavidus*) (Fig. 4B and D).

### OTUs associated with the recipient soils.

**FIG 5:**
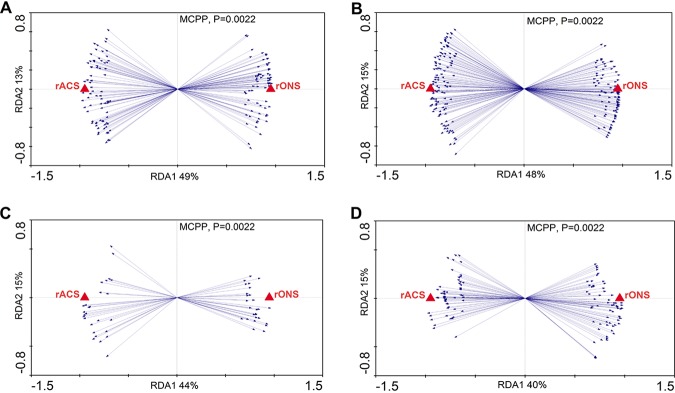
Redundancy analysis (RDA) plot of the bacterial community composition determined by recipient type. The recipient types were used as environmental variables. The OTUs are indicated by blue arrows. *P* values were obtained by the Monte Carlo permutation procedure (MCPP). (A and B) ONS inoculum under aerobic (A) and anaerobic (B) conditions. (C and D) ACS inoculum under aerobic (C) and anaerobic (D) conditions.

The soil bacterial community structure was primarily impacted by the inoculum. However, differences were also detected when the same inoculum was used to seed different soils, indicating that differences in soil physical and chemical properties also influenced the growth of some bacteria. OTUs that were specifically impacted by the recipient ONS (rONS) and ACS (rACS) soils were identified using RDA (Fig. 5). When only the ONS inoculum samples were taken into account, a large number of OTUs were significantly positively impacted by the recipient type (Fig. 5A and B). This finding was confirmed when only the ACS inoculum samples were taken into account (Fig. 5C and D).

## DISCUSSION

Identifying the mechanisms by which bacterial communities are generated and maintained in the soil is essential for understanding the biological functions of soil (2). Numerous studies have attempted to elucidate the main ecological processes by which the microbiota was assembled (4, 24–27). Among all attempts, an approach often used is to investigate the microbiota structures developed in microcosms of which the well-referenced inocula were inoculated into sterilized samples (24, 25). However, significant discrepant conclusions were drawn among previous studies (5, 16, 17). From these seemingly contradictory conclusions arose the question of which factor is more important for the determination of community structure. Here, our results suggest that the inoculum is a more important factor for community assembly. However, we found that inoculum and recipient were demonstrated as the dominant factor for community assembly in sterile recipients because of the varied differences among the inocula used in these experiments.

First, well-designed experiments are necessary to determine the reasons for the variation existing in the explanation of the role of inoculum and environment selection in community assembly. For this purpose, the completeness of the sterility of the recipient soil is an important issue to truly reflect microbial community assembly in the inoculated soil microcosms. In our study, the absence of bacterial and fungal colonies on solidified media after plating diluted irradiated soil subsamples as well as the constant decrease in extracted DNA during incubation of the control microcosms confirmed the effectiveness of the soil sterilization and excluded any bias due to the persistence of surviving cells in the recipient soils. The results also showed that the 2-month incubation time permitted the restoration of a bacterial biomass that was at least quantitatively similar to that estimated in the original soils or in the nonirradiated controls, confirming the development and assembly of a new bacterial community.

According to the perspective of microbial biogeography, the dispersal of microorganisms is limited, and the local species pools in different habitats are dissimilar (28, 29, 53). Dispersal limitation and microbial biogeography can result in different species pools that behave as different inocula immigrating into the recipients. The two inocula from distinct soil habitats in our investigation represented dissimilar local species pools, which determined the bacterial community structuration of the newly developed microbiota. These results indicate that inoculating soil bacteria into sterilized soil microcosms behave similarly when introduced into other habitats, such as liquid media and freshwater microcosms (4, 6, 7), where the assembly of bacterial community in each of these sterilized environments is determined by inoculum. In these cases, the bacteria from the inoculum dominated community assembly (4, 24, 25).

**FIG 6:**
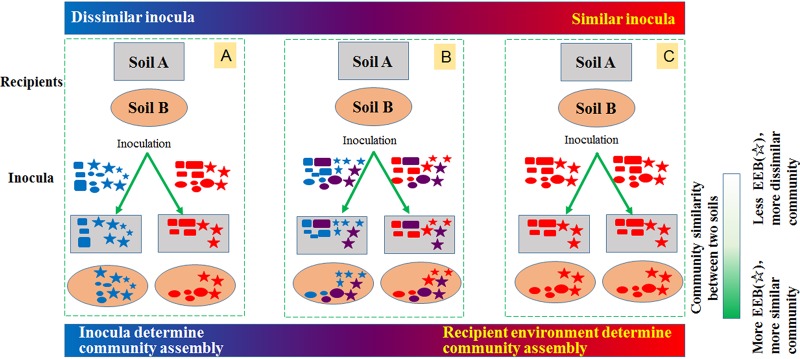
Scheme of community assembly driven by inocula and recipient in soil. (A to C) Community assembly with two inocula containing completely different bacteria (A), with two inocula containing both different and identical bacteria (B), and with two inocula with the identical bacterial community (C). The colorful ovals, rectangles, and stars in the inocula and recipients represent different bacteria. Ecologically equivalent bacteria (EEB) are represented by stars in the scheme. The horizontal axis represents the degree of difference of inocula, either divergence or convergence. The deeper the color, the more dissimilar the inocula. The vertical axis represents the richness of EEB. The deeper the color, the higher the richness of EEB.

Interestingly, some bacterial species in this study acted as indispensable contributors to the developing microbiota and behaved similarly in the two widely different recipient soils (rONS and rACS). These species survived and colonized similarly in an agricultural soil and a telluric environment contaminated by aromatic compounds. For example, 35 genera from the ACS inoculum are known to be common and abundant agricultural soil colonizers (see Text S1 in the supplemental material). In addition, 33 genera (except *Gaiella*) in the ONS inoculum have been reported to possess aromatic- and organic-compound-degrading functions or were detected in sites polluted with aromatic and organic compounds (Text S1). Although these taxa belonged to phylogenetically remote taxa with markedly different functional traits, they exhibited strong involvement in the community reassembly process regardless of the ambient resources available in the two sterilized soil types. The observation that the species exhibited equal fitness in the two environmental conditions is in agreement with the ecological equivalence concept (20, 22). Ecologically equivalent bacteria adapt to different recipient soils; their presence and development in the community are primarily determined by their immigration, although different soils may have different abiotic environments. The prevailing heterogeneous microenvironments inside the soil may create various niches that could provide a wide space to host extremely diverse microorganisms. Thus, the assembly of a microbial community in sterile soil depends on the species immigrating from the exotic environment. The two inocula (ONS and ACS) contained both shared and specific “ecologically equivalent species,” which were not selected by the soil type and proliferated in the recipient soil. They might also be functionally equivalent and perform fundamental biological functions in different soil habitats, such as maintaining the magnitude and stability of nutrient cycling. Thus, they can participate in carbon and nitrogen metabolism in ONS soil or degrade organic pollutants in ACS soil. Most of the “ecologically equivalent OTUs” in our study belonged to the abundant members in the inocula, although others were members of the rare biosphere, such as OTU13 (*Promicromonospora*), OTU15 (unclassified *Fervidicella*), and OTU59 (*Nocardia*). Some of the rare OTUs multiplied rapidly to become predominant in the newly developed soil bacterial communities. We speculate that these rare bacteria were predominant in the history of the source community. The development of equivalent species in the recipient soils led to a significant correlation of the community structure with the inoculum, the most important factor shaping the bacterial community structure. Clearly, when dissimilar ecologically equivalent species immigrated into the recipient soils, they survived or were enriched in sterile environments. Even if the recipient soils were the same, the dissimilar equivalent species provided by the different inocula resulted in distinct community structures. Therefore, the effect of the inoculum on the community structure profile was obvious, and the immigration of equivalent species dominates community assembly in different recipients (summarized in Fig. 6).

10.1128/mSystems.00496-19.4 TEXT S1 Experimental methods and supporting references for ecologically equivalent bacteria. Download Text S1, PDF file, 0.4 MB. Copyright © 2019 Wu et al. 2019 Wu et al. This content is distributed under the terms of the Creative Commons Attribution 4.0 International license.

Although our data demonstrated that the assembly of the bacterial community in soil was determined by bacteria that immigrated from the inocula, the analysis also suggested that the filtering role of the environment was not negligible, particularly when comparing the microbiota developed from one inoculum (either ONS or ACS). In that situation, the finite ecologically equivalent species would occupy corresponding niches in both recipients; however, other nonequivalent species in the inoculum would be differentially selected by the physical and chemical properties of the recipient soils; thus, the niche-adapted bacteria would be selected from the inoculum (4, 24). The sterilized soil provides ambient resources for species immigrating from the inoculum. The aromatic compound contamination in the recipient of ACS (rACS) soil implied that bacteria having specific degrading functions should have higher fitness than other taxa. Similarly, bacteria adapted genetically to agricultural conditions would develop preferentially in the recipient of ONS (rONS) soil. Then, the selection of recipients for nonequivalent species would dominate the differentiation of community assembly, although the different recipients might enrich and share some predominant ecologically equivalent species. Different fertilization treatments in agricultural land that shared the same original species pool equate to the situation of inoculating the similar or same inoculum into different recipients as performed in our investigation. If sterilized soil recipients are inoculated with a similar equivalent bacterial species pool, taxonomic analysis of the resulting microbiota after incubation would indicate the selection of “ecologically nonequivalent species” that colonize specific niches. This finding supports the idea that environmental selection is the determining factor, as shown in a previous study by Xun et al. (5), who reported that the taxonomic structures of the microbiota developing in sterilized microcosms were mainly shaped by the recipient soil properties when the inocula originated from the similar soil source. Under these experimental conditions, the inocula contained similar immigrating species, some of which were ecologically equivalent for all recipients, whereas other nonequivalent species were specifically selected by the specific niches in each soil habitat. Clearly, selection of nonequivalent species by the recipient soils dominated the differentiation of soil microbiota structuration and excluded a role of ecologically equivalent species (summarized in Fig. 6).

According to our results, we found that the community structures are shaped by multiple factors (both inoculated bacteria and environmental factors) (Fig. 3). The findings are consistent with an integrated perspective for the explanation of community assembly, such as the concept of meta-community (30), which emphasizes different processes and their potential importance (31). The local microbiota is considered to be a product of random events during the recruitment of ecologically equivalent microorganisms from the source community or meta-community, which explains why there were differences in the roles of immigrated bacteria and environmental selection for dominating the community assembly processes in previously published studies. The variability in different studies might be caused by the degree of differences among the inocula used for these experiments. The key point for these two factors to establish the determinative role in community assembly might lie in the similarity of species pools that were used for immigration.

In conclusion, we distinguished the relative importance between inoculum and recipient environment in the determination of community structure. We found that inoculum was more important in the process of community assembly. Our investigation also explained the reason for the variability in previous studies about the role of biotic factors and environmental selection. Discrepancy in the determinative factors for community assemblies was associated with the differences between inocula that contained either similar or dissimilar ecological equivalent bacteria. On the basis of the results of our study, we may deduce that once the sources of inoculated bacteria are homologous and their compositions are similar, environmental selection would dominate the assembly processes. If inoculated bacteria are heterologous and phylogenetically dissimilar, they would dominate over the selection effect of environmental conditions. Here, we touched upon the basic and eternal ecological topic about the community-environment interaction. Speculatively, the types of inocula impact the community-environment interactions and thus act as the most important factor for the community assembly. The similarity between inocula is also important for the assembly of the bacterial community. In extreme situations, the inocula have totally different or identical bacteria, as the cases of our experiment with two type of distinctly different inocula or only with one type of inoculum (Fig. 6A and C). However, in many natural situations, the differences of inocula are between above-mentioned situations, in which both inocula and environment selection could be important factors (Fig. 6B). Furthermore, our study showed that there are two types of immigrated bacteria, ecologically equivalent bacteria (EEB) or nonequivalent bacteria, in the inocula. The more ecologically equivalent bacteria are in the inoculum, the more similar bacterial communities assembled in the recipients. The less similar inocula containing much more different ecologically equivalent bacteria would assemble more distinct communities. In that case, the inoculated bacteria would be the predominant factor for determining the bacterial community. Conversely, if the inoculated bacteria are more similar, the assembled bacterial communities would be determined by either ecologically equivalent bacteria or recipient-selected nonecologically equivalent bacteria depending on the community structure of the inoculum (Fig. 6). Therefore, in general, the role of inoculum and recipient environment factors relies on the inocula being used in the experiment.

However, soils are complex environments that dictate the use of artificial conditions to address these questions. The use of sterilized soil microcosms that are artificially reinoculated and incubated under lab conditions is far from a simulation of natural conditions. Therefore, other approaches are required to verify the conclusions drawn in this study. Nevertheless, by using cross inoculation with two distinct soils, we explored the relative importance of inoculum and recipient during community assembly and revealed the reason for the variability in the explanation of the role of these factors. This study would be a step forward toward realizing the prediction and manipulation of the composition and dynamics of naturally occurring microbial communities, which would be beneficial to engineering, medical, agricultural, and environmental science.

## MATERIALS AND METHODS

### Soil sampling.

Two different types of soil were used in this investigation. One was sampled from cultivated farmland with optimized nitrogen fertilization with straw management (optimized nitrogen fertilized soil [ONS]) at a cropland in Quzhou (36°86′N, 115°02′E), Hebei Province, northern China. The field at this sampling site consists of intensively managed agricultural soils, where a winter wheat-summer maize rotation is the dominant crop production system and urea- and NH_4_^+^-based fertilizers are the most commonly applied nitrogen fertilizers (32). The soil is a calcareous fluvo-aquic soil (calcareous Cambisols according to the FAO Classification). The other soil sample (aromatic-compound-contaminated soil [ACS]) was collected from a severely organic-compound-contaminated site at a chemical plant in Shanghai (31°41′N, 121°46′E), eastern China. The parent material of this soil is mainly sediments transported by the Yangtze River that have separated into light loam and medium loam after long-term tillage. The representative characteristic of this ACS soil sample was contamination by polycyclic aromatic hydrocarbons. ONS soil samples were collected from the top 20 cm by sterile manual corers (10-cm diameter), and ACS soil samples were sampled at 150 cm below ground with GeoProbe Systems. Both soil samples were transported and preserved as described previously (5, 17). The two soil samples had distinct physicochemical properties and bacterial community structures, which were reported in our previous study (33).

### Soil microcosms.

After the soil samples were sieved, both subsamples were sterilized by gamma irradiation (50 kGy; Shanghai Heming Radiation Technology Co. Ltd., China), and the irradiated soil samples were stored as in reference 34. The details of the irradiation can be found in the supplemental material (Text S1). To evaluate the efficiency of sterilization, soil suspensions were spread for plate counting with beef extract peptone medium, actinomycetes culture medium, Martin medium, and 1/10 tryptic soy agar medium. Sterile water was added to maintain a constant moisture level (the water content of soil was 19% for ONS and 26% for ACS). The inocula were prepared with nonsterile soil preincubated at 25°C in regularly aerated bags for 1 week. The two sterilized soils (rONS and rACS) were inoculated and mixed with inocula of nonsterile ONS and ACS soils at different inoculation doses (heavy [H] and light [L]) and oxygen conditions (aerobic [ae] and anaerobic [an]) (Text S1). Sterile soil (30 g) was randomly inoculated with one of the inocula at different dosages (0.3 g of soil for the low dose or 3 g of soil for the high dose). In addition, 30 g of nonsterile ONS and ACS soil were incubated in serum bottles as controls. Headspace vials (100 ml;, CNW Technologies, Germany) with soil were sealed to retain moisture. The aerobic incubation bottles were sealed directly in a laminar flow bench (Shanghai, China), and the vials were not supplemented with extra oxygen during the incubation. The gas in the anaerobic incubation vials was replaced with helium by a pumping ventilation system (Shanghai, China). All vials were incubated at 25°C in the dark for 2 months in triplicate for each treatment. There were a total of 20 treatments (60 samples) in this investigation, and each treatment was named according to its management. For example, the four parts of H-ONS-rONS-ae represent the dosage, inoculum, recipient soil, and aeration conditions, respectively. “H” and “L” indicate high- (3 g) or low-dosage (0.3 g) soil inoculum; “ae” and “an” indicate that the soil was incubated under aerobic or anaerobic conditions, respectively; “r” in the recipient indicates that the soil had been sterilized by radiation. To eliminate any effects of exotic soil particles, the soil microcosm was mixed vigorously.

### Analysis of the soil biomass and bacterial community.

DNA was extracted from 0.5 g of soil as described previously (35, 36). Each treatment had triplicate samples, and DNA was extracted from each sample in triplicate to eliminate the influence of soil heterogeneity. The three triplicate extracts of each soil sample were pooled as one DNA sample. The quality of the DNA was assessed based on the 260-nm/280-nm absorbance ratio as measured by a BioDrop μLITE (Biochrom, UK), and the quantity of DNA was measured with a Quan-It PicoGreen double-stranded DNA (dsDNA) assay kit (Invitrogen, USA). Quantitative real-time PCR was conducted on a Light cycler 96 (Roche, Switzerland) using SYBR green as a fluorescent dye to determine the relative abundance of the 16S rRNA gene (37) (Text S1).

The extracted DNA was used as the template to amplify the V3-V4 region of the 16S rRNA gene to construct the sequencing library. The library was constructed according to the manufacturer’s instructions (38) with some modifications as previously described (39) and sequenced on the Illumina MiSeq platform (Illumina Inc., USA) as previously described with minor modifications (40). The preparation of the sequencing library, including DNA extraction and PCR amplification, was conducted as previously described (41). Specifically, the V3-V4 hypervariable region of the bacterial 16S rRNA gene was amplified from the genomic DNA using the universal primer set B341F/B785F (42). The purified amplicons were sequenced using an Illumina MiSeq system (41).

### Bioinformatics and sequencing data analysis.

Both the forward and reverse ends of the read were trimmed at the base which gave a *q* value (false-discovery rate) of less than 20. If a pair of reads had a minimum overlap of 50 bp, they were merged into a complete read, which was retained only if it was longer than 399 bp, and the expected error was no more than 1 (43). The quality-filtered reads were dereplicated into unique sequences and sorted by decreasing abundance, and singletons were discarded. Nonchimeric OTU (operational taxonomy unit) representative sequences were picked afterwards by Uparse’s default (44). Further reference-based chimera detection was performed using UCHIME (45) against an RDP classifier training database (v9) (46) as the reference database. The OTU table was finalized by mapping quality-filtered reads to the remaining OTUs with the Usearch (43) global alignment algorithm at a cutoff of 97%.

The number of high-quality reads was greater than 20,000 for all samples. Therefore, the sequences of each sample were randomly reextracted 20,000 reads per time (1,000 permutations) for rarefaction to equalize the differences in sequencing depth using QIIME (47). Representative sequences for each OTU were subjected to the RDP classifier to determine the phylogeny with a bootstrap cutoff of 80% (RDP database version 2.10). The alpha diversity and beta diversity of the bacterial community structure were calculated with QIIME (47). The phylogenetic tree for the UniFrac analysis (48) was constructed with the maximum-likelihood method by FastTree (49).

The statistical significance of community structural similarity between different clusters was assessed by multivariate analysis of variance (MANOVA) with MATLAB 2014a (MathWorks Inc., Natick, MA, USA) as described by Xu et al. (50) and Tong et al. (51). A redundancy analysis (RDA) performed with CANOCO for Windows 4.5 (Microcomputer Power, Ithaca, NY, USA) was used to investigate the most significant environmental variables that shifted their composition and structure (52). For the RDA, a Monte Carlo permutation test was used to examine the correlations between community structure and each variable (*P* < 0.05). Statistical significance was assessed by the Monte Carlo permutation procedure (MCPP) with 9,999 random permutations under the full model. Variation partitioning analysis (VPA) was performed to partition the total variation of the dependent variable into various portions as described by Xun et al. (5).

### Data availability.

The data on natural ONS and ACS samples have been reported in reference 33.

The 16S rRNA gene sequences in this study were submitted to the GenBank Sequence Read Archive (SRA) database in the National Center for Biotechnology Information (NCBI) under accession number SRP153935.
