# Supplementary material for: Variability in the Response of Bacterial Community Assembly to Environmental Selection and Biotic Factors Depends on the Immigrated Bacteria, as Revealed by a Soil Microcosm Experiment
Source: mSystems. 2019 Dec 3;4(6):e00496-19. doi: 10.1128/mSystems.00496-19 (PMC6890929; doi:10.1128/mSystems.00496-19)
Supplement: TABLE S1 [file mSystems.00496-19-st001.pdf]

Table S1 Quantity of DNA and 16S rRNA gene copy in different treatments

Treatments	DNA quantity（μg/g of soil）	16S rRNA gene (copies/g of soil)[b]
H-ONS-rONS-ae	9.29±3.26[a]	$2.44\times10^{13}\pm1.50\times10^{13}$
H-ONS-rONS-an	13.18±5.45	$1.73\times10^{11}\pm7.90\times10^{10}$
L-ONS-rONS-ae	5.68±2.68	$5.63\times10^{11}\pm6.61\times10^{11}$
L-ONS-rONS-an	34.66±15.48	$4.17\times10^{11}\pm1.80\times10^{11}$
H-ONS-rACS-ae	25.59±21.82	$3.32\times10^{12}\pm2.79\times10^{12}$
H-ONS-rACS-an	2.67±0.35	$2.29\times10^{11}\pm6.12\times10^{9}$
L-ONS-rACS-ae	3.34±2.24	$2.98\times10^{11}\pm1.97\times10^{11}$
L-ONS-rACS-an	11.31±14.81	$5.37\times10^{10}\pm8.51\times10^{9}$
H-ACS-rACS-ae	2.14±0.33	$1.31\times10^{11}\pm1.08\times10^{10}$
H-ACS-rACS-an	1.92±0.56	$2.25\times10^{10}\pm6.44\times10^{9}$
L-ACS-rACS-ae	2.33±1.17	$2.92\times10^{10}\pm1.27\times10^{10}$
L-ACS-rACS-an	2.77±1.85	$2.63\times10^{10}\pm1.61\times10^{10}$
H-ACS-rONS-ae	6.22±3.00	$4.32\times10^{10}\pm2.05\times10^{10}$
H-ACS-rONS-an	2.93±1.73	$1.70\times10^{11}\pm9.89\times10^{10}$
L-ACS-rONS-ae	17.51±3.56	$1.21\times10^{12}\pm2.60\times10^{11}$
L-ACS-rONS-an	3.62±2.12	$1.20\times10^{11}\pm7.96\times10^{10}$
ACS-ae	7.46±5.16	$3.70\times10^{11}\pm2.74\times10^{11}$
ACS-an	5.58±6.16	$8.21\times10^{10}\pm8.83\times10^{10}$
ONS-ae	13.29±9.71	$7.28\times10^{11}\pm5.57\times10^{11}$
ONS-an	17.98±11.19	$1.45\times10^{12}\pm3.15\times10^{11}$

[a]Values (mean ± standard deviation) indicate each index.

[b]Number of 16S rRNA gene copies in a gram soil samples of different treatments.
