# Supplementary figures and images for: Variability in the Response of Bacterial Community Assembly to Environmental Selection and Biotic Factors Depends on the Immigrated Bacteria, as Revealed by a Soil Microcosm Experiment
Source: mSystems. 2019 Dec 3;4(6):e00496-19. doi: 10.1128/mSystems.00496-19 (PMC6890929; doi:10.1128/mSystems.00496-19)

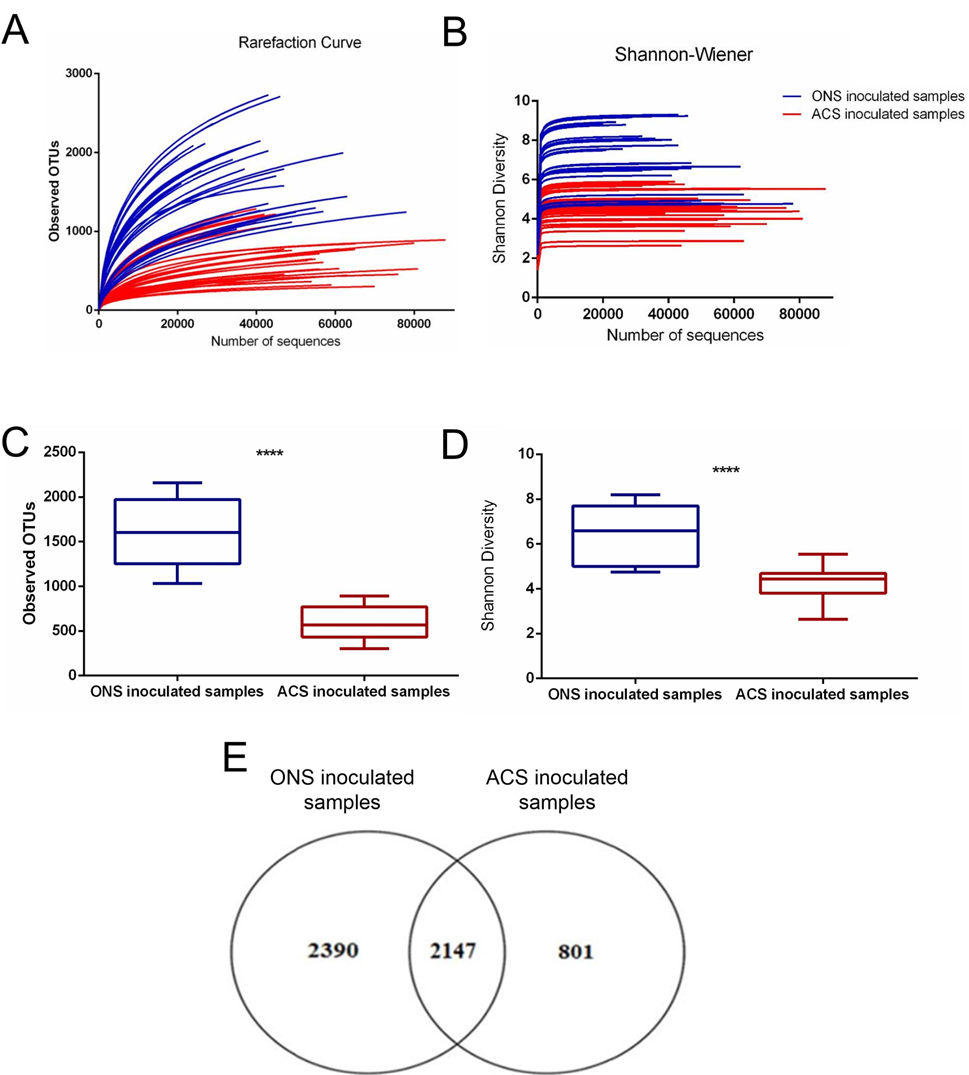

Supplement: FIG S1 [file mSystems.00496-19-sf001.tif]
