# Supplementary material for: Variability in the Response of Bacterial Community Assembly to Environmental Selection and Biotic Factors Depends on the Immigrated Bacteria, as Revealed by a Soil Microcosm Experiment
Source: mSystems. 2019 Dec 3;4(6):e00496-19. doi: 10.1128/mSystems.00496-19 (PMC6890929; doi:10.1128/mSystems.00496-19)
Supplement: TABLE S2 [file mSystems.00496-19-st002.pdf]

Table S2 Alpha diversity indices of different treatments after two months' incubation

Treatments	Observed OTUs[a]	Enspie[b]	Margalef[c]	Menhinick[d]	Shannon[e]	Simpson[f]	PD whole tree[g]
H-ONS-rONS-ae	1839±149[h]	12.64±1.37	109.33±8.87	0.41±0.033	6.61±0.05	0.92±0.009	108.99±7.39
H-ONS-rONS-an	2034±123	56.06±8.89	120.93±7.32	0.45±0.028	7.92±0.16	0.98±0.003	119.19±4.89
L-ONS-rONS-ae	1278±21	10.12±9.39	75.94±1.26	0.29±0.005	5.25±0.84	0.84±0.097	78.03±2.08
L-ONS-rONS-an	1247±98	28.34±1.77	74.1±5.83	0.28±0.022	6.5±0.04	0.96±0.002	79.59±3.72
H-ONS-rACS-ae	1731±92	27.27±7.46	102.91±5.50	0.39±0.021	7.29±0.42	0.96±0.012	103.25±5.13
H-ONS-rACS-an	2052±55	39.51±11.52	122.02±3.28	0.46±0.012	8.1±0.1	0.97±0.007	122.6±2.31
L-ONS-rACS-ae	1207±74	10.48±0.2	71.76±4.40	0.27±0.017	4.87±0.09	0.9±0.002	78.84±4.18
L-ONS-rACS-an	1353±287	7.64±2.08	80.4±17.05	0.3±0.064	5.66±1.03	0.86±0.035	86.16±16.7
H-ACS-rACS-ae	709±70	10.32±1.44	42.09±4.19	0.16±0.016	4.74±0.2	0.9±0.013	50.93±5.14
H-ACS-rACS-an	832±67	15.46±2.53	49.45±4.00	0.19±0.015	5.37±0.28	0.93±0.012	66.5±6.5
L-ACS-rACS-ae	383±124	6.03±0.54	22.74±7.37	0.09±0.028	3.79±0.2	0.83±0.015	30.62±8.06
L-ACS-rACS-an	394±53	10.59±2.92	23.38±3.14	0.09±0.012	4.49±0.17	0.9±0.026	35.07±3.51
H-ACS-rONS-ae	804±43	9.82±1.79	47.77±2.58	0.18±0.01	4.61±0.21	0.9±0.021	54.76±1.41
H-ACS-rONS-an	637±25	5.23±1.49	37.85±1.46	0.14±0.005	3.94±0.26	0.8±0.063	51±1.16
L-ACS-rONS-ae	503±41	9.61±0.28	29.84±2.41	0.11±0.009	4.45±0.03	0.9±0.003	35.53±1.32
L-ACS-rONS-an	432±11	2.89±0.86	25.64±0.64	0.1±0.002	2.97±0.39	0.64±0.098	35.07±1.16
ACS-ae	1086±41	14.17±0.73	64.52±2.44	0.24±0.009	5.75±0.06	0.93±0.004	75.02±1.98
ACS-an	1244±38	10.78±1.78	73.96±2.25	0.28±0.008	5.73±0.22	0.91±0.017	85.43±2.93
ONS-ae	2318±350	188.15±43.09	137.84±20.8	0.52±0.078	8.99±0.23	0.99±0.001	131.18±14.87
ONS-an	2359±389	209.97±40.74	140.26±23.16	0.53±0.087	9.11±0.24	1±0.001	133.12±17.79

[a]Detected OTU (operational taxonomic units) number

[b]Enspie alpha diversity measure.

[c]Margalef's richness index

[d]Menhinick's richness index
[e]Shannon entropy of counts, default in bits.
[f]Simpson's index.
[g]Phylogenetic diversity.
[h]Values (mean ± standard deviation) indicate each index.
