# Supplementary material for: Variability in the Response of Bacterial Community Assembly to Environmental Selection and Biotic Factors Depends on the Immigrated Bacteria, as Revealed by a Soil Microcosm Experiment
Source: mSystems. 2019 Dec 3;4(6):e00496-19. doi: 10.1128/mSystems.00496-19 (PMC6890929; doi:10.1128/mSystems.00496-19)
Supplement: TEXT S1 [file mSystems.00496-19-s0001.pdf]

Experimental methods:

Soil sterilization

The soil was sterilized under the CO_{60} irradiation. In order to avoid the change of the soil physical and chemical properties by irradiation, the dosage of γ-irradiation for each time is not over 50kGy. Subsamples of ONS soil were irradiated by triple times. The first irradiation was performed in September 2014 (36kGy). The second and third time times of irradiation were performed in January 2015 (50kGy for each time), and the interval is 7 days. Subsamples of ACS soil were irradiated by twice (50kGy for each time). The irradiations were performed in January 2015, and the interval is 7 days. To identifying the sterilization, soil suspensions were performed according to spread plate method with beef extract peptone medium, actinomycetes culture medium, martin medium and 1/10 tryptic soy agar medium for microbial identification test.

The determination of soil physical and chemical parameters

Soil properties were determined based on the following standard procedures used in China (1). Soil pH was detected with a pH meter (Mettler-Toledo, Switzerland) in the soil solution at a soil-to-water ratio of 1:2.5. Dissolved organic carbon (DOC) was determined with Elab-TOC (Suzhou Elab analytical instrument Co., Ltd, China). Dissolved nitrogen (DN) was determined by a SHIMADZU TN (Total Nitrogen) Unit. Concentrations of ammonia and nitrate in the extracts were determined by full wavelength spectrophotometry (1). Soil nitrite was measured as described by R. J. Stevens and R. J. Laughlin (2).

Description of treatments in the investigation

Treatment	Description
H-ONS-rONS-ae	3 gram ONS inoculum was inoculated into radiated sterile ONS receptor

	under aerobic condition for 2-month incubation.
L-ONS-rONS-ae	0.3 gram ONS inoculum was inoculated into radiated sterile ONS receptor under aerobic condition for 2-month incubation
H-ONS-rONS-an	3 gram ONS inoculum was inoculated into radiated sterile ONS receptor under anaerobic condition for 2-month incubation.
L-ONS-rONS-an	0.3 gram ONS inoculum was inoculated into radiated sterile ONS receptor under anaerobic condition for 2-month incubation.
H-ONS-rACS-ae	3 gram ONS inoculum was inoculated into radiated sterile ACS receptor under aerobic condition for 2-month incubation.
L-ONS-rACS-ae	0.3 gram ONS inoculum was inoculated into radiated sterile ACS receptor under aerobic condition for 2-month incubation
H-ONS-rACS-an	3 gram ONS inoculum was inoculated into radiated sterile ACS receptor under anaerobic condition for 2-month incubation.
L-ONS-rACS-an	0.3 gram ONS inoculum was inoculated into radiated sterile ACS receptor under anaerobic condition for 2-month incubation
H-ACS-rACS-ae	3 gram ACS inoculum was inoculated into radiated sterile ACS receptor under aerobic condition for 2-month incubation.
L-ACS-rACS-ae	0.3 gram ACS inoculum was inoculated into radiated sterile ACS receptor under aerobic condition for 2-month incubation
H-ACS-rACS-an	3 gram ACS inoculum was inoculated into radiated sterile ACS receptor under anaerobic condition for 2-month incubation.
L-ACS-rACS-an	0.3 gram ACS inoculum was inoculated into radiated sterile ACS receptor under anaerobic condition for 2-month incubation
H-ACS-rONS-ae	3 gram ACS inoculum was inoculated into radiated sterile ONS receptor under aerobic condition for 2-month incubation.
L-ACS-rONS-ae	0.3 gram ACS inoculum was inoculated into radiated sterile ONS receptor under aerobic condition for 2-month incubation
H-ACS-ONS-an	3 gram ACS inoculum was inoculated into radiated sterile ONS receptor under anaerobic condition for 2-month incubation.
L-ACS-ONS-an	0.3 gram ACS inoculum was inoculated into radiated sterile ONS receptor under anaerobic condition for 2-month incubation
ONS-ae	30 gram ONS soil was incubated for 2 months under aerobic condition.
ONS-an	30 gram ONS soil was incubated for 2 months under anaerobic condition.
ACS-ae	30 gram ACS soil was incubated for 2 months under aerobic condition.
ACS-an	30 gram ACS soil was incubated for 2 months under anaerobic condition.

Bioinformatics and sequencing data analysis

The quality of sequencing run was also monitored with mock community controls of known species composition and relative abundance according to suggestions of the manufacturer's

instructions.

Variations for explaining the community structure in VPA analysis include the inoculum (ONS/ACS), aeration type (aerobic and anaerobic), recipient soil (a binary variable of rONS/rACS and the physicochemical soil measurements) and inoculum dosage.

Primers and program used for real-time PCR analysis of the 16S rRNA gene

Gene	Primer	Sequence(5'-3')[a]	Thermal profile
16S	Uni331F	TCCTACGGGAGGCAGCAGT	95℃ for 180s
rRNA	Uni797R	GGACTACCAGGGTATCTAATCCTGTT	40 cycles×(95℃ for 30s,60℃ for 30s, 72℃ for 30s), 80℃ for 5s, data was collected in the last step

Supporting references:

ACS inoculum enriched genera and corresponding references reporting these bacteria in farmland soil

Aeration condition	Genus	References
Aerobic	*Dyadobacter*	(3)
	Phytohabitans	(4)
	Variovorax	(5)
	Promicromonospora	(6)
	Actinosynnema	(7)
	Citricoccus	(8)
	Nitrobacter	(9)
	Methyloversatilis	(10)
	Nocardia	(11, 12)
	Nocardioides	(13)
	Aciditerrimonas	(14)
	Oceanibaculum	(15)
	Pseudomonas	(16, 17)
	Luedemannella	(18)
	Micromonospora	(19)
	Pedomicrobium	(20)
	Arthrobacter	(21)
Anaerobic	*Nocardioides*	(13)
	Micromonospora	(19)

	Aeromicrobium	(13)
	Polaromonas	(22)
	Luedemannella	(18)
	Aciditerrimonas	(14)
	Arthrobacter	(21)
	Aliidiomarina	(23)
	Pseudomonas	(16, 17)
	Fervidicella	(24)
	Cellulomonas	(25, 26)
	Geosporobacter	(27)
	Pelotomaculum	(28)
	Verrucosispora	(29)
	Catellatospora	(30)
	Kitasatospora	(31)
	Marmoricola	(32)
	Janibacter	(33)

ONS inoculum enriched genera and corresponding references reporting these bacteria can degrade aromatic and organic compounds

Aeration condition	Genus	References
Aerobic	*Saccharothrix*	(34)
	Pseudonocardia	(35, 36)
	Aeromicrobium	(37)
	Streptomyces	(38, 39)
	Luteimonas	(40)
	Sphingomonas	(41, 42)
Anaerobic	*Azonexus*	(43-45)
	Ensifer	(46)
	Luteimonas	(40)
	Sphingomonas	(41, 42)

ONS inoculum enriched genera and corresponding references reporting these bacteria are in aromatic and organic compounds enriched sites

Aeration condition	Genus	References
Aerobic	*Blastococcus*	(47)
	Aciditerrimonas	(48)
	Ohtaekwangia	(49)
	Acidobacterium_ Gp4	(50)
	Acidobacterium_ Gp6	(50)
	Gemmatimonas	(51-53)
	Geminicoccus	(54)

	Nitriliruptor	(55)
	Terrimonas	(56, 57)
Anaerobic	*Ohtaekwangia*	(49)
	Microvirga	(50)
	Cupriavidus	(56)
	Lysobacter	(52)
	Geminicoccus	(54)
	Euzebya	(55)
	Gemmatimonas	(51-53)
	Terrimonas	(56, 57)
	Thermosulfurimonas	(58)
	Nitriliruptor	(55)
	Nitrospira	(59, 60)
	Acidobacterium_ Gp4	(50)
	Acidobacterium_ Gp6	(50)
	Acidobacterium_ Gp16	(50, 61)

References list

1. Wu XG, Li J, Ji MM, Wu QY, Wu XX, Ma YM, Sui WK, Zhao LP, Zhang XJ. 2019. Non-synchronous Structural and Functional Dynamics During the Coalescence of Two Distinct Soil Bacterial Communities. Frontiers in Microbiology 10:1125. https://doi.org/10.3389/Fmicb.2019.01125.
2. Stevens RJ, Laughlin RJ. 1995. Nitrite Transformations during Soil Extraction with Potassium Chloride. Soil Science Society of America Journal 59:933-938.
3. Tkacz A, Poole P. 2015. Role of root microbiota in plant productivity. Journal of Experimental Botany 66:2167-2175.
4. Inahashi Y, Matsumoto A, Omura S, Takahashi Y. 2012. Phytohabitans flavus sp. nov., Phytohabitans rumicis sp. nov. and Phytohabitans houttuyneae sp. nov., isolated from plant roots, and emended description of the genus Phytohabitans. International Journal of Systematic and Evolutionary Microbiology 62:2717-2723.
5. Constancias F, Prévost-Bouré NC, Terrat S, Aussems S, Nowak V, Guillemin JP, Bonnotte A, Biju-Duval L, Navel A, Martins JM. 2014. Microscale evidence for a high decrease of soil bacterial density and diversity by cropping. Agronomy for Sustainable Development 34:831-840.
6. Ting S, Tan SH, Meikay W. 2009. Isolation and characterization of actinobacteria with antibacterial activity from soil and rhizosphere soil. Australian Journal of Basic and Applied Sciences 3:4053-4059.
7. Wei W, Isobe K, Nishizawa T, Lin Z, Shiratori Y, Ohte N, Koba K, Otsuka S, Senoo K. 2015. Higher diversity and abundance of denitrifying microorganisms in environments than considered previously. The ISME Journal 9:1954-1965.
8. Matsui T, Semba H, Hanada S. 2012. Citricoccus yambaruensis sp. nov., a racemic phenylsuccinate stereospecifically assimilating actinomycete isolated from soil in Okinawa. Journal of General and Applied Microbiology 58:373-378.
9. Degrange V, Bardin R. 1995. Detection and counting of Nitrobacter populations in soil by PCR. Applied and Environmental Microbiology 61:2093-2098.
10. Badri DV, Zolla G, Bakker MG, Manter DK, Vivanco JM. 2013. Potential impact of soil microbiomes on the leaf metabolome and on herbivore feeding behavior. New Phytologist 198:264-273.

11. Khan ZU, Chugh TD, Chandy R, Provost F, Boiron P. 1998. A study of the enzymatic profile of soil isolates of Nocardia asteroides. Mycopathologia 143:151-154.

12. de Komaid AAV, Duran EL. 1998. Antimicrobial susceptibilities of strains of Nocardia brasiliensis isolated from soil of Tucuman. Mycopathologia 141:115-121.

13. Topp E, Mulbry WM, Zhu H, Nour SM, Cuppels D. 2000. Characterization of S-triazine herbicide metabolism by a Nocardioides sp. isolated from agricultural soils. Applied and Environmental Microbiology 66:3134–3141.

14. Hu L, Cao L, Zhang R. 2014. Bacterial and fungal taxon changes in soil microbial community composition induced by short-term biochar amendment in red oxidized loam soil. World Journal of Microbiology and Biotechnology 30:1085-1092.

15. Liu Y, Zuo S, Xu L, Zou Y, Song W. 2012. Study on diversity of endophytic bacterial communities in seeds of hybrid maize and their parental lines. Archives of Microbiology 194:1001-1012.

16. Green SK, Schroth MN, Cho JJ, Kominos SK, Vitanza-Jack VB. 1975. Agricultural plants and soil as a reservoir for Pseudomonas aeruginosa. Applied Microbiology 28:987-991.

17. Garbeva P, Veen JA, Elsas JD. 2004. Assessment of the diversity, and antagonism towards Rhizoctonia solani AG3, of Pseudomonas species in soil from different agricultural regimes. Fems Microbiology Ecology 47:51-64.

18. Kim JS, Lee KC, Kim DS, Ko SH, Jung MY, Rhee SK, Lee JS. 2015. Pyrosequencing analysis of a bacterial community associated with lava-formed soil from the Gotjawal forest in Jeju, Korea. Microbiologyopen 4:301-312.

19. Hayakawa M, Sadakata T, Kajiura T, Nonomura H. 1991. New methods for the highly selective isolation of Micromonospora and Microbispora from soil. Journal of Fermentation and Bioengineering 72:320-326.

20. Toyota K, Kuninaga S. 2006. Comparison of soil microbial community between soils amended with or without farmyard manure. Applied Soil Ecology 33:39-48.

21. Aislabie J, Bej AK, Ryburn J, Lloyd N, Wilkins A. 2005. Characterization of Arthrobacter nicotinovorans HIM, an atrazine-degrading bacterium, from agricultural soil New Zealand. FEMS Microbiology Ecology 52:279-286.

22. Weon HY, Yoo SH, Hong SB, Kwon SW, Stackebrandt E, Go SJ, Koo BS. 2008. Polaromonas jejuensis sp. nov., isolated from soil in Korea. International Journal of Systematic and Evolutionary Microbiology 58:1525-1528.

23. Xu L, Sun JQ, Wang LJ, Liu XZ, Ji YY, Shao ZQ, Wu XL. 2016. Aliidiomarina soli sp. nov., isolated from saline-alkaline soil. International Journal of Systematic and Evolutionary Microbiology:https://doi.org/10.1099/ijsem.0.001709.

24. Dia A, Lauga B, Davranche M, Fahy A, Duran R, Nowack B, Petitjean P, Henin O, Martin S, Marsac R. 2015. Bacteria-mediated reduction of As(V)-doped lepidocrocite in a flooded soil sample. Chemical Geology 406:34-44.

25. Braun M, Kim JM, Schmid RD. 1992. Purification and some properties of an extracellular l -amino acid oxidase from Cellulomonas cellulans AM8 isolated from soil. Applied Microbiology and Biotechnology 37:594-598.

26. Viamajala S, Smith WA, Sani RK, Apel WA, Petersen JN, Neal AL, Roberto FF, Newby DT, Peyton BM. 2007. Isolation and characterization of Cr(VI) reducing Cellulomonas spp. from subsurface soils: implications for long-term chromate reduction. Bioresource Technology 98:612-622.

27. Morrison JM, Murphy CL, Baker K, Zamor RM, Nikolai SJ, Wilder S, EIshahed MS, Youssef NH. 2017. Microbial communities mediating algal detritus turnover under anaerobic conditions. Peerj 5:https://doi.org/10.7287/PEERJ.PREPRINTS.2453.

28. Salehlakha S, Miller M, Campbell RG, Schneider K, Elahimanesh P, Hart MM, Trevors JT. 2005. Microbial gene expression in soil: methods, applications and challenges. Journal of Microbiological Methods 63:1-19.

29. Lian T, Jin J, Wang G, Tang C, Yu Z, Li Y, Liu J, Zhang S, Liu X. 2017. The fate of soybean residue-carbon links to changes of bacterial community composition in Mollisols differing in soil organic carbon. Soil Biology and Biochemistry 109:50-58.

30. Liu C, Zhao J, Guan X, Li L, Li W, Wang X, Xiang W. 2014. Catellatospora aurea sp. nov., a novel actinomycete isolated from soil. Antonie van Leeuwenhoek 106:1185-1190.

31. Shrivastava S, D'Souza SF, Desai PD. 2008. Production of indole-3-acetic acid by immobilized actinomycete (Kitasatospora sp.) for soil applications. Current Science 94:1595-1604.

32. Dastager SG, Lee JC, Ju YJ, Park DJ, Kim CJ. 2008. Marmoricola bigeumensis sp. nov., a member of the family Nocardioidaceae. International Journal of Systematic and Evolutionary Microbiology 58:1060-1063.

33. Yamazoe A, Yagi O, Oyaizu H. 2004. Degradation of polycyclic aromatic hydrocarbons by a newly isolated dibenzofuran-utilizing Janibacter sp. strain YY-1. Applied Microbiology and Biotechnology 65:211-218.

34. HUYuting, RENFenghua, ZHOUPeijin, XIAMin, LIUShuangjiang. 2003. Degradation of pyrene and characterization of Saccharothrix sp. PYX-6 from the oligotrophic Tianchi Lake in Xinjiang Uygur Autonomous Region, China. Science Bulletin 48:2210-2215.

35. Juteau P, Rho D, Larocque R, Leduy A. 1999. Analysis of the relative abundance of different types of bacteria capable of toluene degradation in a compost biofilter. Applied Microbiology and Biotechnology 52:863-868.

36. Kohlweyer U, Thiemer B, Schräder T, Andreesen JR. 2000. Tetrahydrofuran degradation by a newly isolated culture of Pseudonocardia sp. strain K1. Fems Microbiology Letters 186:301–306.

37. Chaillan F, Le FA, Bury E, Phantavong YH, Grimont P, Saliot A, Oudot J. 2004. Identification and biodegradation potential of tropical aerobic hydrocarbon-degrading microorganisms. Research in Microbiology 155:587-595.

38. Samanta SK, Singh OV, Jain RK. 2002. Polycyclic aromatic hydrocarbons: environmental pollution and bioremediation. Trends in Biotechnology 20:243-248.

39. Balachandran C, Duraipandiyan V, Balakrishna K, Ignacimuthu S. 2012. Petroleum and polycyclic aromatic hydrocarbons (PAHs) degradation and naphthalene metabolism in Streptomyces sp. (ERI-CPDA-1) isolated from oil contaminated soil. Bioresource Technology 112:83-90.

40. Bacosa HP, Inoue C. 2015. Polycyclic aromatic hydrocarbons (PAHs) biodegradation potential and diversity of microbial consortia enriched from tsunami sediments in Miyagi, Japan. Journal of Hazardous Materials 283:689-697.

41. Leys NMEJ, Ryngaert A, Bastiaens L, Verstraete W, Top EM, Springael D. 2004. Occurrence and phylogenetic diversity of Sphingomonas strains in soils contaminated with polycyclic aromatic hydrocarbons. Applied and Environmental Microbiology 70:1944-1955.

42. Chen J, Wong MH, Wong YS, Tam NF. 2008. Multi-factors on biodegradation kinetics of polycyclic aromatic hydrocarbons (PAHs) by Sphingomonas sp. a bacterial strain isolated from mangrove sediment. Marine Pollution Bulletin 57:695-702.

43. Fries MR, Zhou J, Cheesanford J, Tiedje JM. 1994. Isolation, characterization, and distribution of denitrifying toluene degraders from a variety of habitats. Applied and Environmental Microbiology 60:2802-2810.

44. Reinhold-Hurek B, Hurek T. 2006. The genera Azoarcus, Azovibrio, Azospira and Azonexus. Prokaryotes:873-891.

45. Anders H, Kaetzke A, Kämpfer P, Ludwig W, Fuchs G. 1995. Taxonomic Position of Aromatic-Degrading Denitrifying Pseudomonad Strains K 172 and KB 740 and Their Description as New Members of the

Genera Thauera, as Thauera aromatica sp. nov., and Azoarcus, as Azoarcus evansii sp. nov., Respectively, Members of the Beta Subclass of the Proteobacteria. International journal of systematic bacteriology 45:327-333.

46. Muratova A, Pozdnyakova N, Makarov O, Baboshin M, Baskunov B, Myasoedova N, Golovleva L, Turkovskaya O. 2014. Degradation of phenanthrene by the rhizobacterium Ensifer meliloti. Biodegradation 25:787-795.

47. Folwell BD, McGenity TJ, Whitby C. 2016. Biofilm and Planktonic Bacterial and Fungal Communities Transforming High-Molecular-Weight Polycyclic Aromatic Hydrocarbons. Applied and Environmental Microbiology 82:2288-2299.

48. Catania V, Sara G, Settanni L, Quatrini P. 2017. Bacterial communities in sediment of a Mediterranean marine protected area. Canadian Journal of Microbiology 63:303-311.

49. Ma Q, Qu YY, Zhang XW, Shen WL, Liu ZY, Wang JW, Zhang ZJ, Zhou JT. 2015. Identification of the microbial community composition and structure of coal-mine wastewater treatment plants. Microbiological Research 175:1-5.

50. Pérez-Leblic MI, Turmero A, Hernández M, Hernández AJ, Pastor J, Ball AS, Rodríguez J, Arias ME. 2012. Influence of xenobiotic contaminants on landfill soil microbial activity and diversity. Journal of Environmental Management 95:S285-S290.

51. Zhang S, Wan R, Wang Q, Xie S. 2011. Identification of anthracene degraders in leachate-contaminated aquifer using stable isotope probing. International Biodeterioration and Biodegradation 65:1224-1228.

52. Kawasaki A, Watson ER, Kertesz MA. 2012. Indirect effects of polycyclic aromatic hydrocarbon contamination on microbial communities in legume and grass rhizospheres. Plant and Soil 358:169-182.

53. Muangchinda C, Chavanich S, Viyakarn V, Watanabe K, Imura S, Vangnai AS, Pinyakong O. 2015. Abundance and diversity of functional genes involved in the degradation of aromatic hydrocarbons in Antarctic soils and sediments around Syowa Station. Environmental Science and Pollution Research 22:4725-35.

54. Franzetti A, Gandolfi I, Bertolini V, Raimondi C, Piscitello M, Papacchini M, Bestetti G. 2011. Phylogenetic characterization of bioemulsifier-producing bacteria. International Biodeterioration and Biodegradation 65:1095–1099.

55. Peng M, Zi XX, Wang QY. 2015. Bacterial Community Diversity of Oil-Contaminated Soils Assessed by High Throughput Sequencing of 16S rRNA Genes. International Journal of Environmental Research and Public Health 12:12002-12015.

56. Bacosa H, Suto K, Inoue C. 2010. Preferential degradation of aromatic hydrocarbons in kerosene by a microbial consortium. International Biodeterioration and Biodegradation 64:702-710.

57. Singleton DR, Richardson SD, Aitken MD. 2011. Pyrosequence analysis of bacterial communities in aerobic bioreactors treating polycyclic aromatic hydrocarbon-contaminated soil. Biodegradation 22:1061-1073.

58. Païssé S, Goñi-Urriza M, Coulon F, Duran R. 2010. How a bacterial community originating from a contaminated coastal sediment responds to an oil input. Microbial Ecology 60:394-405.

59. Pérezjiménez JR, Young LY, Kerkhof LJ. 2001. Molecular characterization of sulfate-reducing bacteria in anaerobic hydrocarbon-degrading consortia and pure cultures using the dissimilatory sulfite reductase (dsrAB) genes. Fems Microbiology Ecology 35:145–150.

60. Ramos DT, Da SM, Nossa CW, Alvarez PJ, Corseuil HX. 2014. Assessment of microbial communities associated with fermentative-methanogenic biodegradation of aromatic hydrocarbons in groundwater contaminated with a biodiesel blend (B20). Biodegradation 25:681-691.

61. Li X, Wu Y, Lin X, Zhang J, Zeng J. 2012. Dissipation of polycyclic aromatic hydrocarbons (PAHs) in soil microcosms amended with mushroom cultivation substrate. Soil Biology and Biochemistry 47:191-197.
